# Effects of blood flow restriction exercise with very low load and low volume in patients with knee osteoarthritis: protocol for a randomized trial

**DOI:** 10.1186/s13063-019-3238-2

**Published:** 2019-02-18

**Authors:** Mikhail Santos Cerqueira, Wouber Hérickson de Brito Vieira

**Affiliations:** Department of Physical Therapy, Federal University of Rio Grande do Norte, Av. Senador Salgado Filho, 3000 - Campus Universitário, Lagoa Nova, Natal, Rio Grande do Norte 59072-970 Brazil

**Keywords:** Knee pain, Vascular occlusion exercise, Resistance training, Rehabilitation, Muscle strength

## Abstract

**Background:**

Knee osteoarthritis (OA) is characterized by chronic pain, physical dysfunction, and reduced quality of life. Low-load resistance exercises with blood flow restriction (BFR) have presented results similar to those of high-intensity resistance exercise (HIRE) without BFR provided that the exercise volume in both is paired. However, it is unclear whether BFR exercise with reduced load and volume generates clinical improvements similar to those of HIRE. The aim of the proposed study is to evaluate the effects of BFR resistance exercise with very low load and low volume against HIRE in patients with knee OA for the outcomes of knee pain, muscle performance, physical function, disease severity, quality of life, perceived exertion during the exercises, adherence, and patient satisfaction with treatment.

**Methods:**

This two-arm, prospectively registered, randomized controlled trial with blinded assessors and volunteers will involve 40 patients with knee OA. Two weekly treatment sessions will be provided for 12 weeks. Patients will perform very low-load (10% of 1-RM) and low-volume BFR exercise or HIRE (60% of 1-RM) for strengthening thigh muscles. The primary outcome will be the knee pain measured after 12 weeks of treatment. The secondary outcomes include knee pain 6 months after randomization, physical function, disease severity, quality of life, muscle performance, knee pain and perceived exertion during exercise, adherence, and patient satisfaction with treatment.

**Discussion:**

If the improvements in the outcomes are similar in the two groups, BFR exercise with reduced load and volume may be an interesting alternative in the treatment of knee OA, especially when exercises with high loads generate joint pain.

**Trial registration:**

*Registro Brasileiro de Ensaios Clínicos* (REBEC), RBR-6pcrfm. Registered on July 10, 2018.

**Electronic supplementary material:**

The online version of this article (10.1186/s13063-019-3238-2) contains supplementary material, which is available to authorized users.

## Background

Osteoarthritis (OA) is the most common type of arthritis and is characterized by inflammation and major structural changes in the joint [1, 2]. This condition is debilitating because of pain and physical disability, leading to a significant reduction in quality of life [3]. The knee joint is strongly affected by OA, and the weakness of the quadriceps muscle is a major risk factor for knee OA [4].

Strengthening knee extensor muscles is recommended as a key point in reducing pain and disability in these patients [5], and resistance exercises with loads greater than 60% of 1 maximal repetition (1-RM) are classically prescribed to gain strength [6, 7]. However, movements with high loads in the knee with OA can aggravate pain, swelling, and inflammation [8] and consequently reduce adherence to exercise [9]. Resistance exercise with blood flow restriction (BFR) has recently been considered in the treatment of knee OA [10, 11]. BFR resistance exercise is usually performed with lower load (20–30% of 1-RM) combined with a pneumatic cuff inflation that reduces arterial flow and limits the venous return, thus elevating metabolic stimulus in working muscles [12].

BFR exercise may be useful in knee OA treatment because of the possibility of strength gains associated with lower levels of pain, perceived exertion, overload, and joint stress during the training sessions compared with high-intensity (≥60% of 1-RM) resistance exercise (HIRE) without BFR [13–16]. However, it is unclear whether BRF is really required, even in resistance exercises with loads less than 30% of 1-RM [8, 17]. In subjects with knee OA, 8 weeks of resistance training with high (60% of 1-RM) or very low (10% of 1-RM) load without BFR induced similar improvement in pain and muscle function, as the total exercise volume (series × repetitions × load) was paired [8]. In addition, regardless of the load (20% or 50% of 1-RM), exercise volume, or BFR, healthy and untrained individuals presented similar strength increases after 8 weeks of resistance training in which the repetitions were performed until failure [17].

Importantly, in most studies on BFR exercise for knee pain, despite the low load (20–30% of 1-RM), the total volume is very close to [18, 19] or greater than [15, 20] the HIRE volume (~60% of 1-RM) because of a greater number of repetitions in BFR exercise. Thus, it is unclear whether the strength gains promoted by BFR exercise in subjects with knee OA are due to the additional metabolic stimulus promoted by BFR or only to the similar volume when compared with HIRE.

Therefore, this study aims to evaluate the effects of BFR resistance exercise with very low load and low volume against HIRE in patients with knee OA for the outcomes of knee pain, muscular performance, physical function, and quality of life. An additional objective is to identify which exercise protocol will induce lower levels of knee pain and perceived exertion during the exercises and greater adherence and patient satisfaction with treatment.

## Methods

### Study design

**Fig. 1:**
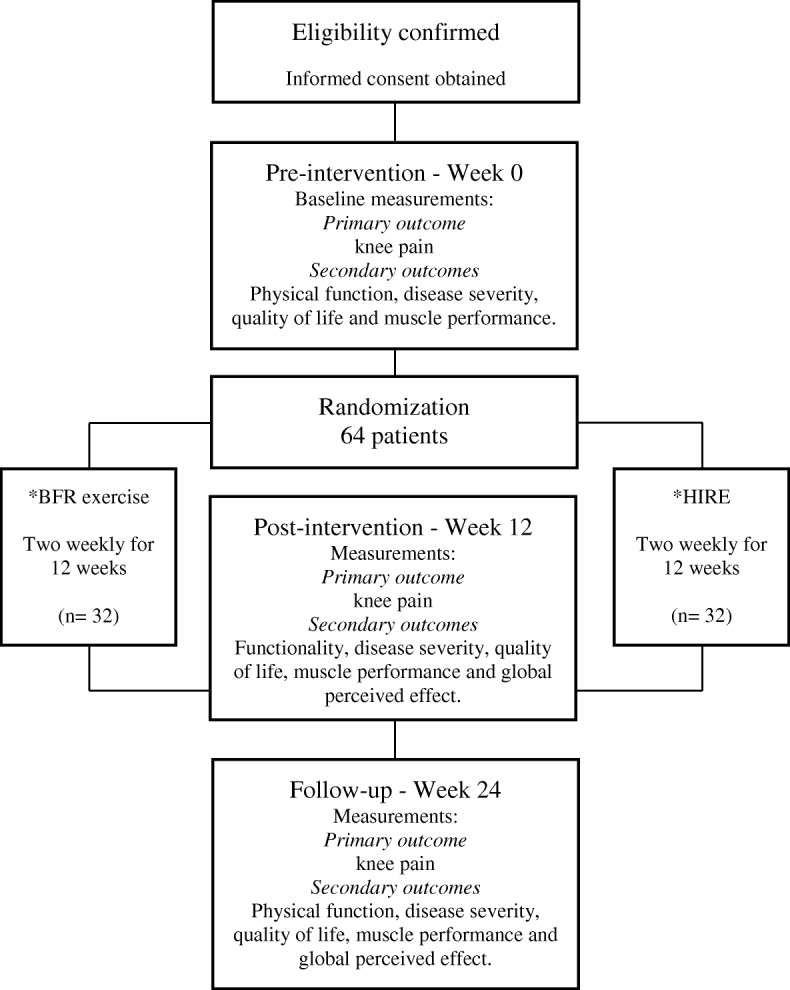
Flow diagram of the planned protocol pathway. Abbreviations: *BFR* blood flow restriction; *HIRE* high-intensity resistance exercise. *During the exercise sessions, the outcomes knee pain, perceived effort, and feelings of pleasure/displeasure will be measured (Feeling Scale)

This is a prospectively registered, two-arm randomized placebo-controlled trial with concealed allocation, blinded measurers and volunteers, intention-to-treat analysis, and 3 months of follow-up (Fig. 1). Subjects with knee OA will be recruited via online media advertisement, personal invitation, telephone, and SMS. Volunteers will be randomly allocated to the BFR exercise group or HIRE group, and the outcome measures will be evaluated in the time points shown in Table 1.

**Table 1:** Time point of outcomes

Outcome measures	Time points														
	Baseline	Weeks of treatment													Follow-up
	W0	W1	W2	W3	W4	W5	W6	W7	W8	W9	W10	W11	W12	MPT	W24
VAS	√													√	√
Lequesne	√													√	√
SF-36	√													√	√
TRP	√			√			√			√				√	√
Functional tests	√													√	√
Isometric and isokinetic tests	√													√	√
7–10 RM test	√			√			√			√				√	√
VAS during exercise		√	√	√	√	√	√	√	√	√	√	√			
Borg		√	√	√	√	√	√	√	√	√	√	√	√		
Feeling Scale		√	√	√	√	√	√	√	√	√	√	√	√		
GPE														√	√

*Abbreviations:* *GPE* Global perceived effect, *MPT* Measures 72 h post treatment, *TRP* Total restriction pressure, *VAS* Visual analog scale, *W* Week

**Fig. 2:**
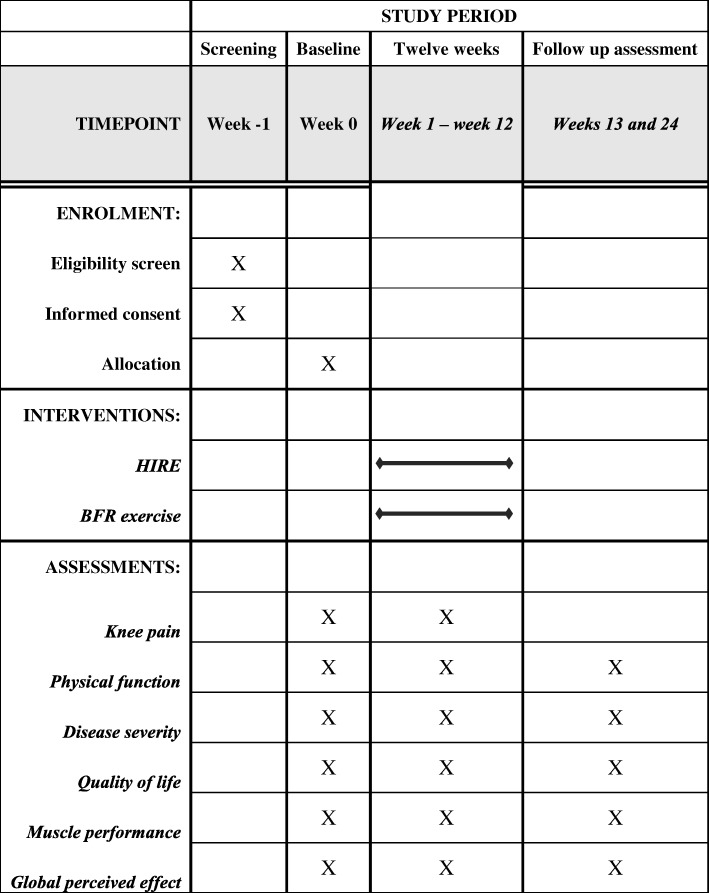
Study design schedule in accordance with the Standard Protocol Items: Recommendations for Interventional Trials (SPIRIT) checklist. Abbreviations: *BFR* blood flow restriction, *HIRE* high-intensity resistance exercise

Analyses of inclusion criteria, getting informed consent, data collection, and statistical analyses will be carried out by researchers blinded to group allocation. Participants will receive oral and written instructions about study risks and benefits and sign a consent form. All personal data will be confidential. The study has obtained ethical approval from the Research Ethical Committee of Universidade Federal do Rio Grande do Norte (CAAE: 91753618.4.0000.5537). The trial was prospectively registered at the *Registro Brasileiro de Ensaios Clínicos* (*RBR-6pcrfm*). All participants will sign an informed consent form prior to participation. The study follows the SPIRIT (Standard Protocol Items: Recommendations for International Trials) 2013 checklist [21] (Fig. 2 and Additionalfile 1) and the TIDieR (Template for Intervention Description and Replication) [22].

### Inclusion and exclusion criteria

A total of 40 men and women with unilateral or bilateral knee OA diagnosed according to the American College of Rheumatology criteria will be considered eligible if they:

- are at least 50 years of age;
- are in the postmenopausal period (for women);
- are between 1.50 and 1.75 m in height;
- have a body mass index between 18.5 and 35 kg/m^2^;
- have moderate to very severe knee OA (score between 5 and 13 on the Lequesne Questionnaire);
- have a score of at least 24 on the Mini-Mental State Examination;
- do not have peripheral vascular disease, systolic blood pressure greater than 160 or less than 100 mm Hg, diastolic blood pressure greater than 100 mm Hg, deep vein thrombosis, diabetes, history of myocardial infarction, stroke in the previous year, or history of cancer that has generated limitations to exercise;
- do not have other orthopedic/neurological diseases that affect gait and do not present any other inflammatory myoarticular disease;
- have not undergone surgery or any invasive procedure on the knees in the previous 6 months;
- have not participated in physiotherapeutic treatment or lower-limb strengthening programs in the past 3 months;
- are not practicing regular physical activity (two or more times per week) for lower limbs (except for those who only practice walking).

Participants will be excluded if they:

- experience pain that completely prevents them from performing the exercises proposed by two consecutive or three non-consecutive sessions or refuse to remain in the study;
- begin taking specific medications that are for OA or that interfere with study outcomes;
- begin to exercise or perform other treatment modalities for knee OA after initiating treatment.

Patients will be able to maintain treatment with stable doses of anti-inflammatory or other medications of regular use in the 3 months prior to the start of the study [15, 23] but should avoid using medications on evaluation days. Subjects who discontinue participation in the study will be invited to participate in the assessments 3 months after the start of treatment, and 6 months after the randomization. Thus, all individuals will be included in the intention-to-treat analysis. Therapists who deliver the intervention will be eligible if they have undergone specific training for the treatment protocol.

### Randomization

Volunteers included will be randomly allocated to BFR exercise or HIRE groups by using the website www.randomization.com. Balanced permutations in blocks with respect to the presence of unilateral or bilateral knee OA will be used. Participant allocation will be concealed in sequentially numbered and sealed opaque envelopes prepared prior to the study by a research assistant, who will not be involved in the study.

### Intervention

The intervention will consist of two weekly sessions with about 60 min of duration each during 12 weeks for a total of 24 sessions. The volunteers will be re-evaluated after 12 weeks and from then on will be guided to practice exercises according to their own volition [20]. In both groups, the sessions will be started with 5 min of light-intensity warm-up on a stationary bike. All subjects included will be instructed not to initiate regular exercises beyond treatment sessions [8, 18]. Both groups will perform bilateral strengthening of the thigh muscles through squatting exercises on the hack machine (angle between 0° and 60° of knee flexion) and leg extension exercises (angle between 90° and 45° of knee flexion) [11, 20]. These amplitudes of movement were selected to minimize the load of the patellofemoral joint during the exercises [24].

In addition to exercises to strengthen the thigh muscles, trunk, hip, and calf exercises will be performed for both groups. High- or low-intensity exercises for strengthening thigh muscles are strongly recommended [25]. Although other studies have focused only on strengthening thigh muscles [8, 11, 18, 20], we chose an overall treatment because of the pathophysiology of knee OA [26] and because there is strong evidence that associating knee exercise and training of proximal and distal muscles to this joint is effective in patients with knee pain [27, 28]. The detailed exercise protocol is shown in Table 2.

**Table 2:** Treatment protocol performed by the high-intensity resistance exercise group and blood flow restriction exercise group (adapted from Bryk et al. [15], 2016)

High-intensity resistance exercise group	
- Hamstring stretching, three repetitions of 30 s	
- Bridge with isometric contraction of the transversus abdominis, CORE training, three repetitions of 30 s ^+^	
- Hip abduction with weights (lying on side), three sets of 10 repetitions ^+^	
- Calm exercises (lying on side) with elastic band, three sets of 10 repetitions ^+^	
- Calf raises, three sets of 10 repetitions ^+^	
- Sensori-motor training (standing) on a mini-trampoline, three repetitions of 30 s	
- Squats on a hack machine, 0°–60° of knee flexion, three sets of eight repetitions *	
- Seated knee extensions (machine), 90°–45° of knee flexion, three sets of eight repetitions *	
Blood flow restriction exercise group	
- Hamstring stretching, three repetitions of 30 s	
- Bridge with isometric contraction of the transversus abdominis, CORE training, three repetitions of 30 s ^+^	
- Hip abduction with weights (lying on side), three sets of 10 repetitions ^+^	
- Calm exercises (lying on side) with elastic band, three sets of 10 repetitions ^+^	
- Calf raises, three sets of 10 repetitions ^+^	
- Sensori-motor training (standing) on a mini-trampoline, three repetitions of 30 s	
- Squats on a hack machine, 0°–60° of knee flexion, one set of 30 repetitions and three sets of 15 repetitions ^‡^	
- Seated knee extensions (machine), 90°–45° of knee flexion, one set of 30 repetitions and three sets of 15 repetitions ^‡^	

The interval between sets will be 30 s and between exercises will be 2 min

*Load is 60% of the 1-repetition maximum

^+^The load will be adjusted every 3 weeks to maintain an effort perception between 6 and 7 on the Borg scale

^‡^Load is 10% of the 1-repetition maximum

Loads of 10% and 60% 1-RM in the BFR exercise and HIRE groups, respectively, will be estimated (1-RM estimated) from the maximum load that can be overcome in 7–10 repetitions (7–10 RM test) [20] based on the Brzycki equation, (W/(1.0278–0.0278 × R), where W refers to weight used in the repetitions until failure and R refers to repetitions to failure [29, 30]. The load will be readjusted every 3 weeks with a 2- to 4-day interval after the last treatment session to prevent residual exercise fatigue from interfering with the 7–10 RM test. Five minutes of warm-up will be performed on a stationary bike before the 7–10 RM test [20, 31]. In relation to the 1-RM test, the 7–10 RM test has the advantage of minimizing the effect of pain on maximum force generation [20].

Participants will be advised that knee pain or discomfort during exercise is normal and that this does not necessarily cause joint damage [9]. All exercises should be performed with pain levels between 0 and 5 on the visual analog scale (VAS) defined as acceptable pain. The session will be interrupted if the patient reports pain greater than 5 or is unable to complete the exercise [23]. The load will be reduced by 20% (relative to 60% of estimated 1-RM) in the HIRE group if the pain prevents the volunteers from completing the exercise [20].

#### Blood flow restriction exercise

The BFR exercise group will perform the squatting on the hack machine and leg extension combined with a BFR corresponding to 60% of total restriction pressure (TRP) in both thighs [20]. The TRP will be individually determined to generate similar metabolic stimulus between participants [32]. After 10 minutes of rest in a climatized room (between 23° and 25 °C), the TRP will be determined with volunteers positioned in supine decubitus with their upper and lower limbs relaxed. The transducer (5 to 10 MHZ) of a portable vascular Doppler (DV 2001, MEDPEJ, Ribeirão Preto, Brazil) will be positioned at the ankle at a mean distance between medial malleolus and calcaneus tendon to capture the auscultatory signal of the posterior tibial artery. A manufactured pneumatic cuff (10 cm width and 80 cm length) will be positioned on the proximal end of the thigh [31, 33] and inflated based on a previous protocol [34]. The TRP will be readjusted every 3 weeks.

The same cuff used in the TRP evaluation will be inflated in the proximal end of the thigh immediately before the exercises (squatting in the hack machine and extension of the leg) and will remain inflated during the interval rest between sets. The cuff will be removed during the 2-min interval between exercises. Fluctuations in prescribed pressure (60% of TRP) will be monitored and regulated by the therapist [18]. The number of repetitions completed in each series will be monitored to check whether the total proposed volume was reached. The total duration of the BFR will be about 5 min per exercise.

The load during the squatting exercises on the hack machine and leg extension will be 10% of the estimated 1-RM [8] with 1 × 30 and 3 × 15 repetitions to be performed with a 30-s interval between sets [14, 15, 18, 20]. The total volume (load × repetitions × series) for each exercise per session will be estimated 1-RM × 0.1 × 75 = estimated 1-RM × 7.5 kg.

#### High-intensity resistance exercise

The HIRE group will perform 3 × 8 repetitions with 60% 1-RM for squatting on the hack machine and leg extension exercises [8] with an interval of 30 s between repetitions. Total volume (load × repetitions × series) for each exercise per session will be 1-RM estimated × 0.6 × 24 = 1-RM estimated × 14.4 kg. In the HIRE group, a BFR placebo with the same cuff used in the BFR exercise group but inflated with a minimum pressure (10 mm Hg) will be applied. This BFR level will not affect the number of repetitions per session [20].

### Primary outcome

The primary outcome is knee pain at rest and during the 30-s chair stand test and will be measured by using the VAS from 0 to 100 mm, where “0 mm” means no pain and “100 mm” means maximum pain already experienced.

### Secondary outcomes

Secondary outcomes are knee pain 3 months after the end of treatment, physical function, disease severity, quality of life, muscle performance, knee pain and effort perceived during exercises, patient adherence, and satisfaction with treatment.

Knee pain 3 months after the end of treatment will be measured by VAS, as described in the primary outcome.

Physical function will be evaluated through tests recommended by the Osteoarthritis Research Society International: 30-s chair stand test, 40-m fast-paced walk test, stair climb test, and timed up-and-go test [35].

Disease severity will be measured by Lequesne’s algofunctional questionnaire, a tool composed of 10 questions regarding pain, discomfort, and function. The sum of the scores is classified as little (1–4 points), moderate (5–7 points), severe (8–10 points), very severe (11–13 points), and extremely severe (≥14 points) dysfunction [36].

Quality of life will be evaluated through the SF-36 questionnaire, a tool composed of 36 items regarding functional capacity, physical aspects, pain, general health, vitality, social aspects, emotional aspects, and mental health. The total score varies from 0 to 100, and higher indexes are related to better quality of life [37].

Bilateral muscle performance measures will be the 7–10 RM test, isometric and isokinetic torque, and rate of torque development (RTD) of knee flexors and extensor muscles. Participants will be instructed to report any pain during the assessments during these measures and whether pain has prevented them from achieving maximum strength [20]. The 7–10 RM test will be performed as previously described to determine the estimated 1-RM.

Isometric and isokinetic torque will be evaluated by isokinetic dynamometer (Biodex Multi-Joint System 3, Biodex Medical System Inc., Shirley, NY, USA) and conducted by a trained researcher. To minimize the possible influence of fatigue on muscle performance, there will be at least a 2-day interval between the 7–10 RM test and torque assessments. A 5-min warm-up will be performed before the evaluations on a stationary bicycle followed by a pre-test (three isometric and isokinetic sub-maximal repetitions). The volunteers will be positioned in the isokinetic dynamometer chair following the recommendations of the equipment manufacturer and with the dynamometer rotation axis aligned with the rotation axis of knee joint (lateral femoral epicondyle), and the torque evaluations will be initiated on the non-affected side (or on the less affected side for subjects with bilateral OA).

Isometric torque will be evaluated first. The subjects will perform three maximal isometric actions with the knee flexed at 60° for extensor analysis and 35° for knee flexor analysis (0° = total extension). Volunteers will be previously and carefully guided with standardized verbal stimulation to contract as fast and strong as possible after the command “go”, hold the contraction for 5 seconds, and relax after the command “stop”. Six attempts (three for the flexors and three for the extensors) will be performed in each limb with a 30-s interval between attempts [38]. Two minutes after isometric evaluation, the volunteers will remain positioned for concentric isokinetic evaluation (five repetitions with 60°/s speed) of the knee flexor and extensor muscles [18, 19]. Volunteers should extend and flex the knee in a pre-established range of motion (90° to 10°). The RTD will be extracted from the isometric torque curve of evaluated muscles. RTD provides physiological information such as the role of neural and muscular factors in producing strength and neuromuscular fatigue [39, 40]. Analysis of the rate of force development will be conducted as previously proposed [38, 39].

Knee pain during exercise will be assessed at all sessions (immediately after each series) by the VAS. Volunteers will be questioned regarding anterior knee pain during the last five repetitions of each series and will be informed that this pain assessment has no relation to occlusion discomfort or perceived exertion [15]. An average will be calculated to obtain the level of pain per session.

Perceived effort will be evaluated in all of the sessions through the modified 10-point Borg scale [41] immediately before starting the hack machine squatting and leg extension exercises (for baseline recording) and after the last thigh muscle–strengthening exercise. Adherence to treatment will be assessed by calculating the percentage of sessions completed by each volunteer [20].

The participants’ satisfaction with treatment will be inferred from the global perceived effect (GPE) scale and from the sensation of pleasure/displeasure during exercises. The GPE scale evaluates the clinical change perceived by the patient, comparing the onset of symptoms with the last days. This numerical scale consists of 11 points (from −5 to +5: −5, extremely worse; 0, without modification; +5, fully recovered). Higher scores indicate better recovery of the condition [42, 43]. The pleasure/displeasure related to the squatting exercise on the hack machine and leg extension will be evaluated by the Feeling Scale, a scale of 11 points, ranging from −5 (much displeasure) to +5 (much pleasure). The Feeling Scale will be applied once per session immediately after the last thigh muscle–strengthening exercise, immediately after perceived effort evaluation. The outcome measures will be conducted by the same evaluator throughout the study, and all equipment will be calibrated before starting the study.

### Masking/blinding

Participants will be masked for the intervention they receive and will be instructed not to talk about their experience during the exercise if they incidentally encounter other participants. Furthermore, the study interventions and measurements will occur in separate locations and the treatment sessions will be individualized, thus facilitating the blinding of participants and evaluators. Participants from both groups will receive the TRP evaluation to induce the placebo effect and will be informed that BFR during exercise is effective in increasing muscle strength and reducing knee pain.

### Sample size estimates

It is estimated that a sample size of 32 patients per group would be necessary to test our research hypothesis on the primary outcome (knee pain). The parameters utilized to sample size calculation included an intergroup variability (σ) in the VAS gain of 21% for both groups [44]; a non-inferiority limit (d) of 14%, which is less than the minimum clinically perceptible difference, usually considered to be 15% for the VAS [45]; a type-I error of 5% (α =0.05); a power of 80% (β=0.20) and a dropout rate of 15%.

### Statistical analyses

The data distributions of normality will be evaluated by the Kolmogorov–Smirnov test. In the case of normal distribution, an analysis of covariance (ANCOVA test) with Tukey’s post hoc will be used to test the differences between groups. The between-group mean difference, 95% confidence interval, and effect size (Cohen’s f) will be reported. The Kruskal–Wallis test will be used for non-normal data distribution, and the effect size will be calculated with Cohen’s r. All participants will be included in the analysis following an intention-to-treat approach.

## Discussion

This trial will examine whether BFR resistance exercise with very low load and low volume will present effects similar to those of the HIRE on pain, muscular performance, physical function, disease severity, and quality of life in patients with knee OA. Although previous studies have shown positive effects of BRF exercises, the true efficacy of this intervention is hampered by the matching of the total exercise volume compared with HIRE [14]. In addition, no study has evaluated the effects of BFR exercise with very low load (10% of 1-RM) and low volume in patients with knee OA. If effective, BFR exercise with 10% of 1-RM can be an interesting treatment for generating lower joint overload and consequently lesser pain during exercise.

The present study can be considered of high methodological quality because it is randomized and prospectively registered, masks the evaluators and patients, concealed allocation, and used an intention-to-treat approach. Sample size was calculated to provide adequate statistical power to identify possible differences in the study primary outcome. In addition, the 3-month follow-up will enable verification of the persistent effects of BFR exercise in patients with knee OA, thus filling this gap since studies on this topic generally assess the effects only immediately after treatment.

The exercise load will be periodically adjusted, thus maintaining the overload principle of strength training. We will estimate the 1-RM from a submaximal test (7–10 RM test) to minimize the pain interference in the load determination. In addition, to avoid a possible measurement bias (gain strength due to learning with the test and not due to neuromuscular adaptations) we choose to measure the strength in three different ways (isometric, isokinetic and 7-10 RM test). [46]. Another strength of this trial is external validity. In order to enable extrapolation of the study findings to a larger portion of the population, it was decided not to limit the participation of patients according to gender, unilateral or bilateral involvement, and the use (or not) of medications. The randomization will be balanced and the statistical analysis will be adjusted according to these factors to minimize possible confounding factors due to this greater sample coverage.

This protocol is not free of limitations. First, the percentage of occluded blood flow will not be measured; instead, the percentage relative to the TRP will be calculated; therefore, it is not possible to guarantee the exact amount of BFR. Furthermore, the TRP will be measured at rest, and therefore it will not be possible to guarantee that the amount of restricted blood flow will be the same during exercise since the muscle hemodynamics may be altered because of the action of the muscular pump and release of vasoactive substances. Another limitation of the study is the impossibility of masking the therapist. In conclusion, the results of this trial may indicate the very-low load and low-volume BRF exercise as an effective treatment for knee OA, generating lower joint pain during exercise and increasing adherence and satisfaction with the treatment.

## Trial status

At the time of manuscript submission, the volunteers were being recruited.

## Additional file

Additional file 1: SPIRIT (Standard Protocol Items: Recommendations for Interventional Trials) 2013 Checklist: Recommended items to address in a clinical trial protocol and related documents. (DOC 122 kb)

## Abbreviations

1-RM: 1 maximal repetition
BFR: Blood flow restriction
GPE: Global perceived effect
HIRE: High-intensity resistance exercise
OA: Osteoarthritis
RTD: Rate of torque development
TRP: Total restriction pressure
VAS: Visual analog scale
